# A comparative study of postnatal depression and associated factors in Gauteng and Free State provinces, South Africa

**DOI:** 10.4102/phcfm.v14i1.3031

**Published:** 2022-09-30

**Authors:** Kebogile Mokwena, Perpetua Modjadji

**Affiliations:** 1 Department of Public Health, School of Health Care Sciences, Sefako Makgatho Health Sciences University, Pretoria, South Africa

**Keywords:** postnatal depression, Edinburgh postnatal depression scale, free state, Gauteng, South Africa

## Abstract

**Background:**

The factors contributing to probable postnatal depression (PND), a type of clinical depression that can affect woman after childbirth, are socially derived. Therefore, variations among groups of women necessitate studies in different communities.

**Aim:**

This study compared the prevalence of PND and associated factors among women attending postnatal services facilities.

**Setting:**

The study setting included Tshwane Municipal district in Gauteng province (GP) and Fezile Dabi District (FS) in Free State province (FSP), South Africa.

**Methods:**

A total of 477 mothers within 12 weeks of giving birth were recruited by convenient sampling in health facilities. A self-developed questionnaire was used to obtain information on socio-demographics, obstetric history, and children’s characteristics. The Edinburgh Postnatal Depression Scale (EPDS) was used to collect data on depression symptoms, with a score of ≥ 13 used as a cut-off for probable PND. Data were analysed using STATA 14. Multivariate logistic regression was used to determine association between probable PND and various covariates.

**Results:**

The overall mean age of women was 28 ± 6 years. The overall prevalence rate of PND was 22%, slightly higher in FS (23%) than in GP (21%). Most participants living in GP were married, had tertiary education, were employed and from the households with income of more than R8000.00. A chi-square test showed that planned pregnancy was significantly higher in GP compared with FS (*p* ≤ 0.001). Multivariate logistic regression showed that support from a partner or husband decreased the odds of a probable PND in GP (adjusted odd ratio [AOR] 0.37; 95% confidence interval [CI] [95%CI: 0.14–0.96; *p* = 0.041] and in the FS [AOR = 0.14, 95%CI: 0.05–0.40; *p* ≤ 0.001]). Significant associations of probable PND with several factors – planned pregnancy, baby age, support in difficult times, partner or husband drinking alcohol and stressful events – were more common in the FSP than in the GP.

**Conclusion:**

The prevalence of probable PND and its associated risk factors in the GP and the FS indicates the need for routine screening and targeted interventions in both urban and rural settings.

**Contribution:**

The results confirm that the prevalence of PND is similar in both rural and urban areas, and that pregnancy planning remains a challenge in the FS, which calls for increased efforts to revive family planning programmes in primary health care facilities.

## Introduction

Maternal and child health are priority areas for public health interventions globally, including in South Africa.^1^ However, the targets to reduce maternal and child morbidity and mortality have been consistently missed in South Africa, with mortality rates for children having increased during the Millennium Development Goals (MDGs) period in 1990.^2^ Mental disorders are the largest contributors to disease burden among women of childbearing age,^1^ and poor maternal mental health often impacts the overall health of their children. Although the focus of postnatal depression (PND) is on the mother, it also has a wide range of neurodevelopment consequences for children,^3,4,5,6,7^ including language development.^8^ If left undiagnosed and therefore untreated, PND has long-term negative outcomes on the well-being of the mother and her child.

Postnatal depression affects some women in the immediate postpartum stage, thereby impacting the ability of the mother to care for herself, her baby, and her family.^9^ It has also been linked directly to lower the infant height and weight, higher rates of malnutrition and stunting, higher rate of diarrheal diseases, infectious illnesses, frequent hospital admissions, reduced completion of recommended schedules of immunisations in children, as well as poor cognitive, social, behavioural and emotional development of the child, disturbances in the mother-infant relationship, the mother’s insensitive engagement with the infant, and mortality in children up to 5 years.^10,11^

Although the prevalence of PND varies widely and has been reported to be high globally,^1^ it has a more severe impact on low-and middle-income countries (LMICs).^12^ This is because mental health matters have been neglected in these countries, and PND remains undetected and underdiagnosed for many women as it is not routinely screened.^13^ In developed countries, the prevalence rate of PND ranges from 5% to 74%, reported among women in their initial days of postpartum and using the cut-off score of 9 out of 10 on the Edinburgh Postnatal Depression Scale (EPDS).^14^ In contrast, the prevalence rates of PND in developing countries varied from 2% to 82% using two-item Patient Health Questionnaire (PHQ-2) among presumably healthy women aged 18 – 45 years, who gave birth to a single baby.^15^ Some studies have found PND to be associated with maternal age, being single, lack of income and adverse life events.^16,17^ As adverse life events and coping skills vary, there is a need to conduct studies in specific areas because of the variability associated with the context of studies. Not only is the relationship of the baby’s father relevant for the development and severity of postnatal depression but also the quality of other social relationships, including the lack of a close, trusting relationship with their mothers, as well as lack of support and difficult relationships with in-laws.^18,19^ Antenatal exposure to extreme societal stressors, which includes witnessing a violent crime and being in danger of being killed, difficulties with partners, and the father’s negative attitude towards the infant, has been found to be associated with PND in South Africa.^20^

Although the negative impact of PND on both mother and child is well documented,^21^ this area of maternal mental health has not enjoyed adequate attention in many countries, including South Africa.^22^ The symptoms of PND can be easily identified by the use of the EPDS, a well-validated screening tool that is used in many countries.^23^ Although this tool is economical and easy to use, PND is not routinely screened for in primary healthcare settings in South Africa. This is because the emphasis of maternal and child health priority in South Africa is on decreasing physical morbidity and mortality.^24^ The implications are not only the lack of the estimation of PND but also that the majority of child-bearing Black South African women, who receive their postpartum care at primary healthcare, are neither screened nor treated for PND.

There are contradictory findings regarding the association between PND and rural settings, with some studies reporting an association between rural living as a risk factor for PND,^25,26^ while others have reported that rural residence could be a protective factor for developing psychiatric illness.^27^ This is because in rural communities, where social networks are stronger than in urban settings, women often use family and friends as support structures, and such structures are always available.^27^ This highlights the need for continuing studies among women from both rural and urban settings in South Africa, which may eventually provide a convergence of conclusions.

The prevalence of PND in South Africa has been reported to be higher than in other African countries,^28^ which may be partially explained by a range of reported risk factors experienced by South African women. The prevalence of PND in South Africa varies across communities and socio-economic status. While the prevalence of PND may be as high as 34.7% in a peri-urban area,^29^ a recent study reported a higher prevalence of 50.3% in a rural setting.^30^ In a recent study conducted among adolescents in Kwa Zulu Natal, PND was reported to be as low as 9.0%,^31^ which contrasts with a high of 49.3% reported in Tshwane among an older sample.^13^ Moreover, PND has been reported to be higher among women living with the human immunodeficiency virus (HIV).^32,33,34^ In a population with high HIV prevalence; it implies that many of the women who are not screened, diagnosed, and treated for PND are deprived of a service that is so crucial for them and their children. Children exposed to HIV also have higher vulnerability to developmental delays, which could be exacerbated by mother’s PND.^35^

Because of the wide variations in prevalence estimates in different communities and because South Africa does not have frequently routinely collected data on PND, which would provide country estimates, it is necessary to conduct studies in different areas of the country. This study aimed to compare the prevalence of PND and its associated factors among women attending primary healthcare clinics in Gauteng province (GP) and Free State province (FSP), South Africa, to advocate for better screening procedures.

## Research methods and design

### Study design, population and setting

A cross-sectional survey was conducted among women who had delivered a live infant within 12 weeks of data collection. The participants were attending postnatal care at health facilities in GP and FSP in South Africa. Thirteen health facilities formed part of the study; six were located in GP (Tshwane Metropolitan Municipality) and seven in Fezile Dabi District (FS).

Gauteng is the smallest of South Africa’s provinces in terms of size, although it is one of the most densely populated.^36^ While FS is the third-largest province in South Africa, yet has the second-smallest population and the second-lowest in population density.^37^ The population densities in Gauteng is approximately 809 people per km^2^, while in FS, it is 22.8.^38^ Gauteng still comprises the largest share of the South African population, with approximately 15.81 million people (26.3%) living in this province.^39^ The FS has an estimated population of 2.87 million of which about 80% are dependent on public health services.^40^ Gauteng generated just over a third of South Africa’s gross domestic product (GDP) in 2017, making it the nation’s biggest provincial economy. In addition, Gauteng contributed R1.59 trillion to the country’s GDP of R4.65 trillion, translating to R1 111 71.00 per person. This makes it the top ranking province in terms of GDP per capita, followed by two provinces which include FS.^41^ The differences in the context of these two provinces warranted a comparative analysis of the prevalence of probable PND and associated factors among postpartum women attending the primary health facilities.

### Sample size and sampling techniques

A sample size was calculated using Rao software size calculator.^42^ A study population of 3200 women who delivered in these facilities within the past year was estimated for the two provinces. A sample size calculation considered 5% margin of error and 95% CI to calculate a minimum sample of 344 participants. The district municipalities were selected randomly, as well as the facilities. The study used postpartum women (i.e. purposive sample) and a final sample of 477 was obtained, with 240 women from the GP and 237 from the FSP. The difficulty in obtaining a random sample of mothers waiting in queues for services in the facilities informed the use of selecting women using a convenience sampling. Mothers who met the inclusion criteria mentioned above were enrolled in the study based on their availability and accessibility at the time of the study.

### Data collection, tool and recruitment

Data were obtained from mothers who had delivered a live infant within 12 weeks of the time of data collection, and were attending postnatal care in the identified health care facilities. Mothers were individually recruited as they were waiting to be attended to by health professionals for their postnatal check-up, as well as for well-baby clinic. Upon agreeing to participate, detailed information about the purpose and value of the study and how the results will be used was given. The participants were encouraged to ask questions or clarifications about the study, which was followed by a request to provide informed consent. The questionnaire was administered by trained research assistants in a private room allocated by the facility manager. Demographic data collected included personal information (i.e. age, marital status, education level and employment status) and household information, such as living arrangements, income and child care grant, as well as obstetric history and children’s characteristics.

The EPDS, a globally validated tool for screening for PND across cultures,^23,43,44^ was used to collect data on the mood of women about the previous 2 weeks after their baby was born, as well as health problems, relationship with husband or partner, and history of intimate partner violence (IPV), social support and life stress. The EPDS is reported to have a high sensitivity of 74% (correctly identifying true cases), a specificity of 74% (correctly identifying people who do not have the condition) and its positive predictive value (the proportion of respondents who scored high on the EPDS and whose depression was confirmed by a clinical interview) was reported to be a high of 73%.^45^ Most researchers use a cut-off range of 11 to 13, while few have used a cut-off of 10.^46^ In this study, a cut-off of > 13 was used to indicate PND which excluded those with scores of 11 and 12. The advantage of using a higher cut-off of 13 is that it increases the likelihood of identifying probable PND.^47,48^

The validity in the study was enhanced by the use of the EPDS, which is an already valid, reliable, and standardised tool for screening of PND.^22^ The EPDS tool has been used in previous South African studies.^13,30,49^ Nonetheless, the tool was validated through content and face validity and a pilot study. Content validity was achieved by making use of experts in the field to ensure that the questionnaire items would adequately measure the constructs they were intended to assess, and whether the items were sufficient to measure the area of interest. Face validity was ensured through experts’ judgement to make sure that the questionnaire would measure what it intended to measure. Independent translators who speak IsiZulu and Setswana as their mother tongue and are conversant with English did forward and backward translations of the questionnaire. To pre-test the tool and determine its feasibility, a pilot study was conducted among 15 mothers (3%) of the calculated minimum sample size) in one of the facilities that did not form part of the study, and the results were not included in the data analyses for the main study. Attending to the logistical challenges which were identified during the pilot study resulted in a smooth process during data collection of the main study.^22^

### Data analysis

Raw data were captured on excel spreadsheet and exported to STATA version 15 for analysis. Complete case analysis was used to identify participants with missing data during analysis. Questionnaires with more than 10% missing data were excluded from the study (i.e. three questionnaires), and questionnaires with less than 10% missing data were used in data analysis. Descriptive statistics (i.e., frequency [*n*] and percentage [%]) was computed. A score of 13 and above was used to classify women with and without significant PND symptoms. A Chi-square test was used to compare the sociodemographic status between women with and without PND symptoms; presented as *n* and %. Fisher’s exact was applied to variables with expected values less than 5 in a cell. Univariate and multivariate logistic regression analyses were used to determine the association of PND symptoms with covariates. A purposeful selection process began with a univariate analysis of each variable. Any variable having a significant univariate test at some arbitrary level (*p* < 0.200) was selected as a candidate for the multivariate analysis. Results are presented as adjusted odds ratio (AOR) (95% CI). A significance level of *p* < 0.050 was applied.

### Ethical considerations

Ethical clearance of the study was obtained from the Research and Ethics Committee of Sefako Makgatho Health Sciences University (SMUREC), (No. SMUREC/H/101/2016:IR), as well as from the Provincial Ethics Committees of the two provinces. Permission to conduct the study was obtained from the management of the health facilities, and written consent was obtained from all participants who were above 18 years old. For those aged below 18 years, a written informed consent was sought from their caregivers, and if caregivers were not available, they were excluded from the study. If, during the interview, a participant expressed emotional distress or showed signs of distress, the researcher was ready to stop with the interview and refer the participant to the South African Depression and Anxiety Group (SADAG) who can be reached telephonically between 08:00 and 20:00. The research assistants provided all participants with the contact information of SADAG for future needs. All participants were given information brochures on PND.

## Results

### Socio-demographic characteristics of mothers

As shown in Table 1, the sociodemographic variables of mothers are discussed. The overall mean age of mothers was 28 ± 6 years, with the mean age for GP being 27 ± 6 years, ranging from 16 to 43, while for FSP mean age was 28 ± 6 years ranging from 13 to 44 years. Significant differences of marital status (*p* < 0.001), education level (< 0.001), employment (< 0.001), household income (< 0.001), receiving child care grant (< 0.001), and father’s financial support (*p* = 0.003) were observed between GP and the FSP. Although the sociodemographic status was poor, many women who were ever married, with tertiary education, employed and household income of more than R8000.00 were living in the GP compared with the FSP. The FSP was characterised by significant proportions of mothers with primary education, cohabiting, unemployed, living in households with income of R2001.00 – R5000.00, receiving child care grant, and father’s financial support.

**TABLE 1:** Sociodemographic characteristics of participants.

Variables	Combined provinces		GP		FSP		*p*-value
	*n*	%	*n*	%	*n*	%	
**Age (years)**							0.583
< 30	311	65	159	67	152	64	-
≥ 30	165	35	80	33	85	36	-
**Marital status**							< 0.001*
Single	187	39	87	36	100	42	-
Cohabiting	150	31	54	23	96	41	-
Ever married	140	29	99	41	14	17	-
**Education level**							< 0.001*
No school	5	1	4	2	1	1	-
Primary	180	38	55	23	125	53	-
High	226	47	122	51	104	44	-
Tertiary	66	14	59	24	7	2	-
**Employment**							< 0.001*
Unemployed	334	70	151	63	183	77	-
Student	14	3	4	2	10	4	-
Self-employed	13	3	9	4	4	2	-
Employed	116	24	76	31	40	17	-
**Living with**							0.246
Alone	4	0.8	1	0.4	3	1.3	-
Family	472	99	239	99.6	233	98.3	-
Friends or Other	1	0.2	0	0	1	0.4	-
**Household income**							< 0.001*
Do not know	97	21	64	27	33	14	-
< R500	11	2	2	1	9	4	-
R501 – R1000	34	7	7	3	27	11	-
R1001 – R2000	71	15	23	10	48	20	-
R2001 – R5000	106	22	45	19	61	26	-
R5001 – R8000	66	14	38	16	28	12	-
> R8000	90	19	59	25	31	13	-
**Receiving child care grant**							< 0.001*
No	299	63	168	71	131	55	-
Yes	175	34	69	29	106	45	-
**Father’s financial support**							0.003*
No	64	13	43	18	21	9	-
Yes	413	87	194	82	219	91	-

*n*, number of participants; %, proportion; GP, Gauteng province; FSP, Free State province;

* , significant difference.

### Characteristics of mothers and babies

Most babies in GP were aged between 2 and 4 weeks compared with the FSP (*p* < 0.001). Planned pregnancy was significantly high in the GP compared with the FSP (*p* < 0.001, Table 2).

**TABLE 2:** Mothers’ obstetric history and baby characteristics.

Variables	Combined provinces		GP		FSP		*p*-value
	*n*	%	*n*	%	*n*	%	
**Parity**							0.449
1–2	332	70	173	72	159	67	-
3–4	128	27	60	25	68	29	-
≥ 5	17	3	7	3	10	4	-
**Baby’s age**							< 0.001*
< 1 week	57	12	2	1	55	23	-
2–4 weeks	188	40	127	53	61	26	-
5–8 week	134	28	62	26	72	31	-
≥ 9 weeks	97	20	49	20	48	20	-
**Delivery method**							0.134
Vaginal	331	69	159	66	172	73	-
Caesarean	146	31	81	34	65	27	-
**Planned pregnancy**							< 0.001*
No	263	55	111	46	152	64	-
Yes	214	45	129	54	85	36	-
**Baby’s health**							0.968
Good	439	92	221	92	218	92	-
Sometimes not well	38	8	19	8	19	8	-
**Baby’s gender**							0.009*
Boy	247	52	110	46	137	58	-
Girl	230	48	130	54	100	42	-
**Preferred gender**							0.118
Boy	158	33	73	30	85	36	-
Girl	210	44	103	43	107	45	-
None	109	23	64	27	45	19	-
**Breastfed**							0.484
No	55	12	25	11	30	13	-
Yes	418	88	211	89	207	87	-

*n*, number of participants; %, proportion; GP, Gauteng province; FSP, Free State province;

* , significant difference.

### Factors associated with the likelihood of developing postnatal depression

Table 3 shows the prevalence of probable PND and comparison of categories of exposure to PND across provinces. The overall prevalence rate of probable PND in the study was 22%. The FSP (23%) had a slightly high prevalence rate of PND compared with GP (21%), although not significantly different (*p* = 0.657). The results further showed a significant difference of who support participants in difficult times between the two provinces (*p* = 0.002). Support came mostly from family members.

**TABLE 3:** Comparisons of categories of exposure (risk) factors to postnatal depression across provinces.

Variables	Combined provinces		GP		FSP		*p*-value
	*n*	%	*n*	%	*n*	%	
**Probable PND**							0.657
No	370	78	187	79	183	77	-
Yes	104	22	50	21	54	23	-
**Do you have a partner or husband currently?**							0.430
No	41	9	23	10	18	8	-
Yes	435	91	216	90	219	92	-
**Partner or husband support**							0.785
No	48	11	25	11	25	11	-
Yes	392	89	216	90	196	89	-
**Partner or husband has other sexual partners**							0.765
No	353	77	174	77	179	78	-
Yes	104	23	53	23	51	22	-
**Receiving social support in difficult times**							0.698
No	53	11	28	12	25	11	-
Yes	424	89	212	88	212	89	-
**Who supports you in difficult times**							0.002*
None	44	9	28	12	16	7	-
Family	342	73	172	72	170	74	-
Extended family	23	5	4	2	19	8	-
Friends or others	61	13	36	15	25	11	-
**Partner or husband violent**							0.168
No partner	41	9	23	10	18	8	-
Never	387	81	197	82	190	80	-
Once	18	4	6	3	12	5	-
Few times	20	4	11	4	9	4	-
Many times	10	2	2	1	8	3	-
**Partner or husband drinks alcohol**							0.119
Never	169	38	96	43	73	32	-
Sometimes	209	46	95	42	114	51	-
Everyday	11	2	4	2	7	3	-
Every weekend	61	14	29	13	32	14	-
**Stressful life events**							0.233
No	274	58	144	60	130	55	-
Yes	202	42	95	40	107	45	-

*n*, number of participants; %, proportion; GP, Gauteng province; FSP, Free State province;

* , significant difference.

The crude odds ratio (OR), 95% CI, and *p*-values for the association of PND with covariates by province are presented in Table 1-A1, Table 2-A1 and Table 3-A1. In overall, univariate analysis showed association (*p* < 0.200) of PND with method of delivery, baby planned, baby gender, breastfeeding, partner support, family support, partner having other sexual partners, currently having a partner or husband, partner drinking alcohol and stressful events (see TABLE 1-A1). For GP, univariate analysis showed association (*p* < 0.200) of PND with the method of delivery, place of delivery, baby planned, baby gender, partner support, currently having a partner or husband, partner drinking, and stressful events (see TABLE 2-A1). For FSP, univariate analysis showed association (*p* < 0.200) of PND with baby planned, partner support, family support, partner having other sexual partners, currently having a partner or husband, partner drinking alcohol and stressful events (see TABLE 3-A1).

The AOR, 95% CI and *p*-values for the association of PND with covariate by provinces are presented in Table 4. After controlling for all independent variables (in combined provinces), mothers who received support from partners or husbands were less likely to have PND than those who did not receive support [AOR = 0.28, 95%CI: 0.14–0.55; *p* < 0.001]. Mothers who had partners/husband drinking alcohol every weekend [AOR = 2.1, 95%CI: 1.00–4.56; *p* = 0.050] and drinking alcohol sometimes [AOR = 1.96, 95%CI: 1.10–3.51; *p* = 0.023] were more likely to have PND than mothers who had partners/husbands who were not drinking. Stressful event [AOR = 2.21, 95%CI: 1.34–3.63; *p* = 0.002] increased the odds of having PND compared to mothers who had not experienced stressful event. Support from a partner or husband was the common factor in GP [AOR = 0.37, 95%CI: 0.14–0.96; *p* = 0.041] and FSP [AOR = 0.14, 95%CI: 0.05–0.40; *p* < 0.001] associated with decreased having PND. In the FSP, several factors were associated with PND than in GP. Such factors were planned pregnancy [AOR = 0.39, 95%CI: 0.16–0.95; *p* = 0.039], baby age of 5 to 8 weeks [AOR = 0.32, 95%CI: 0.10–0.98; *p* = 0.046] and ≥ 9 weeks [AOR = 0.19, 95%CI: 0.05–0.73; *p* = 0.015], support in difficult times [AOR = 0.31, 95%CI: 0.11–0.89; *p* = 0.030] and stressful event [AOR = 4.14, 95%CI: 1.87–9.18; *p* < 0.001].

**TABLE 4:** Association between postnatal depression and covariates for combined provinces and across individual provinces (i.e. GP and FSP).

Combined provinces	AOR	95%CI	*p*-value
**Support from partner or husband**			
No	-	-	-
Yes	0.28	0.14–0.55	< 0.001
**Partner or husband drinking alcohol**			
No	-	-	-
Everyday	3.29	0.69–15.69	0.136
Every weekend	2.13	1.00–4.56	0.051*
Sometimes	1.96	1.10–3.51	0.023*
**Stressful event**			
No	-	-	-
Yes	2.21	1.34–3.63	0.002*
**Gauteng province (GP)**			
**Support from partner or husband**			
No	-	-	-
Yes	0.37	0.14–0.96	0.041*
**Partner or husband drinking alcohol**			
No	-	-	-
Everyday	7.84	0.46–135.00	0.156
Every weekend	2.56	0.91–7.19	0.076
Sometimes	1.93	0.87–4.28	0.105
**Free State province (FS)**			
**Support from partner or husband**			
No	-	-	-
Yes	0.14	0.05–0.40	< 0.001*
**Planned pregnancy**			
No	-	-	-
Yes	0.39	0.16–0.95	0.039*
**Baby age (week)**			
< 1	-	-	-
2–4	1.60	0.61–4.25	0.342
5–8	0.32	0.10–0.98	0.046*
≥ 9	0.19	0.05–0.73	0.015*
**Support in difficult times**			
No	-	-	-
Yes	0.31	0.11–0.89	0.030*
**Stressful event**			
No	-	-	-
Yes	4.14	1.87–9.18	< 0.001*

AOR, adjusted odds ratio; CI, confidence interval;

* , significant association.

## Discussion

The current study compared the prevalence of PND and its associated factors among women attending primary healthcare clinics in GP and FSP, South Africa. The overall prevalence rate of PND in this study was 22%. Furthermore, the study showed that the prevalence of PND was slightly high in the FSP (23%) than in the GP (21%), with no statistically significant differences observed between the districts. The factors associated with PND included planned pregnancy, support from a partner or husband baby age, support in difficult times and stressful events, which were more observed among mothers in the FSP than in the GP. In addition, significant differences in the socio-demographic variables were observed between the two provinces, with better living conditions in the GP compared with the FSP.

The prevalence of PND recorded in this study Is comparable with several studies conducted in South Africa^50^ and in other African countries.^51,52^ This prevalence is higher than 16.4% previously reported in the last decade,^53^ but lower than 49.30%, 50.30% and 57.14% recently reported.^13,22,30^ The GP is highly urbanised compared with the FSP, known to have a greater proportion of rural communities. The literature documents that women in rural areas may be at greater risk of PND than their urban counterparts. For instance, a study conducted in rural areas of South Africa reported a 50.30% prevalence of PND^30^ against 39.50% in urban areas.^54^ In some countries, such as Zimbabwe, the prevalence rate of 21.40% for PND has been reported in the rural areas, while in the urban areas a prevalence range of 30.00% – 34.00% has been reported during the postnatal period.^25,52,55^ In addition to settings variation, the discrepancies could be because of the different cut-off points applied to determine PND using the EPDS.

The consequences of PND transcend beyond the mother, therefore, including the family and communities, helps to understand the factors that place postnatal women at risk of PND.^25,56^ As with classical depression, a wide range of social pressures are major contributors to PND.^50^ Many women who were ever married, with tertiary education, employed and with household income of more than R8000.00 live in the GP compared with the FSP. The FSP was characterised by significant proportions of mothers with primary education, cohabiting, unemployed, living in households with income of R2001.00 – R5000.00, receiving child care grant and relying on father’s financial support. For example, lack of financial support from the father of the baby is a risk factor to PND is similar to findings of other studies.^50,57,58^ This lack of financial support also contributes to household poverty, which on its own is a risk factor for PND.^1,12^ The high rates of unemployment in this study contribute to compromised financial situation in a similar way.

In the FSP, several factors were associated with PND than in GP, which included planned pregnancy, baby age, support in difficult times and stressful events. The current study showed that planned pregnancy was significantly high in GP compared with the FSP. Nonetheless, 46.0% of mothers in the GP and 64.0% in the FSP still did not plan their pregnancy. In the FSP, mothers who have planned pregnancy were less likely [AOR = 0.39, 95%CI: 0.16–0.95] to have PND, while in GP there was no association with planned pregnancy. South African studies have reported that unplanned pregnancies are associated with PND.^13,30^ What is concerning in the current study is that 55.2% of mothers (46.0% in the GP and 64.0% in the FSP) did not plan their babies. This confirms the high rates of unplanned pregnancies in South Africa,^59^ which is an indication of weaknesses of the family planning programme in South Africa. Suggestions on improving family planning include involving men.^60^ Unplanned pregnancies were also associated in South Africa with antenatal depression, which is one of the strongest predictors of postnatal depression.^61^

Multivariate analysis in the current study showed that support from partners or husbands was associated with the decreased odd of having PND than those who did not receive support. Support from a partner or husband was the common factor in GP [AOR = 0.37, 95%CI: 0.14–0.96] and FSP [AOR = 0.14, 95%CI: 0.05–0.40] associated with a lesser likelihood of having PND. Therefore, having support from spouses was protective against the likelihood of developing PND in the current study. A lack of support has been implicated in the development of PND in South Africa^1,34^ and other countries,^62,63,64^ predisposing to stress.^65^

Stressful event [AOR = 2.21, 95%CI: 1.34–3.63] increased the odds of having PND in mothers compared with those who had not experienced stressful events in this study. As with other studies,^53,66^ the experience of severe stressful event was significantly associated with PND. Such stressful events included witnessing a violent crime, threat to life, divorce and losing a job. The impact of such stressful events is experienced more severely among people of low socioeconomic status, who do not enjoy adequate access to counselling and other treatment services for mental challenges.^67^ To add to stressful events, mothers who had partners or husbands drinking alcohol every weekend and drinking alcohol sometimes were more likely to have PND than those who had partners or husbands who were not drinking. A high prevalence of alcohol use in the country^68,69^ exposes many women, some who are pregnant or having recently given birth, to violence, which results in poor mental health status. This finding is of particular concern because of the high prevalence of intimate partner violence in South Africa, even among pregnant women, thus increasing the odds of depression.^70^

### Limitations

Firstly, this study was conducted in the health facilities, which makes it difficult to generalise the results to the community. Secondly, the cross-sectional nature of the study meant that all women were assessed for PND only once and could not establish a causal relationship. Future studies could adopt a longitudinal approach, which can track the development of PND from the antenatal period. Thirdly, although purposive sampling was relevant to identify women in the postpartum period in this study, and use of convenient sampling (based on their accessibly and availability), use of non-probability sampling has a tendency of introducing bias and inhibits drawing inferences about a population. Fourthly, we acknowledge a slight variation of a sample size as a limitation in the study, therefore, to avoid the possibility of reducing the power of the study and increasing the margin of error, we could not remove participants with missing data of less than 10% following complete case analysis. Fifthly, we acknowledge the limitation of not testing the reliability of the questionnaire statistically, as one of the characteristics of a valid questionnaire.^71^ Nonetheless, EPDS is a reliable and valid tool that has been validated in South Africa^72^ and used in several studies in the context of South Africa.^13,22,50^ Finally, as with all screening studies, the study was limited to identifying depressive symptoms, and could not verify the depression, which can only be carried out through clinical assessment. However, this limitation is mitigated by the high sensitivity, high specificity and high positive predictive value of the EPDS,^45^ which has been able to estimate the likelihood of PND among postpartum mothers and compared between the two provinces.

## Conclusions

We conclude that the prevalence of PND among women in the two provinces (GP and FSP) of South Africa are high comparable with some studies,^50,52,55^ which warrants context-specific intervention. PND-associated factors included the protectiveness of support from a partner or husband, planned pregnancy, baby age, support in difficult times and the predisposing stressful events. Several associated factors to PND were observed in the FSP than in the GP. The failure of the South African health system to routinely screen for PND in primary healthcare facilities remains a cause for concern, especially because the cohort of such women is exposed to a variety of social pressures that are risk factors for developing PND. Moreover, with limited services for promotion of mental health, many women with PND are never diagnosed and/or treated, with long-term negative outcomes for themselves and their children, thus leading to a failure to support the improvement of maternal and child health treatment outcomes. We recommended that universal screening for PND in primary healthcare facilities be integrated in the package of postnatal care services, which will provide access to basic mental healthcare services for women who depend on such services for their well-being.

The study showed that support from a male partner or husband was the common factor in the two provinces, which reduced the odds of probable PND. Although the inclusion of male partners in the prevention and treatment intervention might offer a holistic approach to caring for women who are experiencing a probable PND, the evidence remains underdeveloped, as suggested by Noonan et al.^73^

## Appendix 1

**TABLE 1-A1:** Univariate analysis (unadjusted odd ratio) on the association of PND and covariates for combined Gauteng and Free State provinces.

Combined provinces	OR	95% CI	*P*-value
**Baby age**			
< 1 week	-	-	-
2–4 weeks	1.08	0.54–2.14	0.834
5–8 weeks	0.60	0.28–1.29	0.191
≥ 9 weeks	0.75	0.34–1.64	0.468
**Delivery method**			
Vaginal	-	-	-
Caesarean	1.38	0.87–2.18	0.175
**Place of birth**			
Home			
Clinic	0.43	0.04–4.87	0.498
Hospital	1.20	0.13–10.89	0.869
**Baby planned**			
No	-	-	-
Yes	0.46	0.29–0.74	< 0.001
**Baby health**			
Good	-	-	-
Sick	1.11	0.51–2.43	0.787
**Baby gender**			
Girl	-	-	-
Boy	1.41	0.91–2.20	0.120
**Baby gender preferred**			
No preference	-	-	-
Boy	0.90	0.50–1.64	0.734
Girl	0.99	0.56–1.72	0.958
**Breastfed**			
No	-	-	-
Yes	0.66	0.35–1.23	0.188
**Partner support**			
No	-	-	-
Yes	0.22	0.12–0.41	< 0.001
**Partner having other sexual partners**			
No	-	-	-
Yes	1.90	1.15–3.13	0.012
**Family support**			
No	-	-	-
Yes	0.59	0.31–1.12	0.108
**Partner violence**			
No partner	-	-	-
Never	0.33	0.17–0.65	< 0.001
Once	0.60	0.18–2.01	0.408
Few times	1.91	0.65–5.63	0.241
Many times	1.56	0.39–6.27	0.529
**Currently having partner or husband**			
No	-	-	-
Yes	0.40	0.20–0.78	0.007
**Partner drinking alcohol**			
No	-	-	-
Everyday	5.45	1.53–19.40	0.004
Every weekend	2.96	1.57–5.98	0.002
Sometimes	1.89	1.09–3.30	0.023
**Stressful event**			
No	-	-	-
Yes	2.69	1.72–4.21	< 0.001

**TABLE 2-A1:** Univariate analysis (unadjusted odd ratio) on the association of PND and covariates for Gauteng Province.

Gauteng province (GP)	OR	95% CI	*P*-value
**Baby age**			
< 1 week	-	-	-
2-4 weeks	0.90	0.41-1.95	0.788
5-8 weeks	0.59	0.23-151	0.275
≥ 9 weeks	omitted	-	-
**Delivery method**			
Vaginal	-	-	-
Caesarean	2.27	1.20-4.31	0.012
**Place of birth**			
Home	-	-	-
Clinic	0.32	0.13-1.10	0.075
Hospital	omitted	-	-
**Baby planned**			
No	-	-	-
Yes	0.54	0.29-1.01	0.057
**Baby health**			
Good	-	-	-
Sick	0.68	0.19-2.44	0.556
**Baby gender**			
Girl	-	-	-
Boy	1.66	0.89-3.12	0.112
**Baby gender preferred**			
No preference	-	-	-
Boy	0.56	0.25-1.27	0.167
Girl	0.62	0.30-1.31	0.213
**Breastfed**			
No	-	-	-
Yes	0.67	0.26-1.71	0.402
**Partner support**			
No	-	-	-
Yes	0.39	0.15-0.99	0.047
**Partner having other sexual partners**			
No	-	-	-
Yes	1.47	0.70-3.071	0.307
**Family support**			
No	-	-	-
Yes	0.93	0.35-2.44	0.879
**Partner violence**			
No partner	-	-	-
Never	0.31	0.12-0.77	0.011
Once	1.56	0.26-0.47	0.632
Few times	1.30	0.30-5.54	0.726
Many times	1.56	0.09-28.14	0.765
**Currently having partner or husband**			
No	-	-	-
Yes	0.37	0.15-0.92	0.031
**Partner drinking alcohol**			
No	-	-	-
Everyday	6.15	0.70-47.70	0.082
Every weekend	2.34	0.86-6.39	0.096
Sometimes	1.74	0.82-3.70	0.151
**Stressful event**			
No	-	-	-
Yes	1.67	0.89-3.13	0.109

**TABLE 3-A1:** Univariate analysis (unadjusted odd ratio) on the association of PND and covariates for Free State Province.

Free State province (FS)	OR	95% CI	*P*-value
**Baby age**			
< 1 week	-	-	-
2-4 weeks	1.43	0.64-3.21	0.387
5-8 weeks	0.59	0.25-1.39	0.227
≥ 9 weeks	0.50	0.18-1.37	0.177
**Delivery method**			
Vaginal	-	-	-
Caesarean	0.80	0.40-1.61	0.530
**Place of birth**			
Home	-	-	-
Clinic	collinearity	-	-
Hospital	collinearity	-	-
**Baby planned**			
No	-	-	-
Yes	0.38	0.18-0.78	0.008
**Baby health**			
Good	-	-	-
Sick	0.68	0.19-2.44	0.556
**Baby gender**			
Girl	-	-	-
Boy	1.63	0.59-4.53	0.345
**Baby gender preferred**			
No preference	-	-	-
Boy	1.67	0.65-4.53	0.289
Girl	1.80	0.73-4.58	0.195
**Breastfed**			
No	-	-	-
Yes	0.65	0.28-1.51	0.316
**Partner support**			
No	-	-	-
Yes	0.13	0.06-0.33	< 0.001
**Partner having other sexual partners**			
No	-	-	-
Yes	2.41	1.21-480	0.012
**Family support**			
No	-	-	-
Yes	0.39	0.17-0.93	0.035
**Partner violence**			
No partner	-	-	-
Never	0.35	0.13-0.98	0.046
Once	0.31	0.05-1.88	0.205
Few times	0.14	0.59-16.8	0.181
Many times	1.57	0.29-8.42	0.598
**Currently having partner or husband**			
No	-	-	-
Yes	0.43	1.58-1.17	0.098
**Partner drinking alcohol**			
No	-	-	-
Everyday	5.33	1.02-27.81	0.047
Every weekend	3.72	1.36-10.22	0.011
Sometimes	2.10	0.92-4.79	0.077
**Stressful event**			
No	-	-	-
Yes	2.26	2.26-8.56	< 0.001

## References

[1] Kathree T, Selohilwe OM, Bhana A, et al. Perceptions of postnatal depression and health care needs in a South African sample: The ‘mental’ in maternal health care. BMC Womens Health. 2014;14:140. 10.1186/s12905-014-0140-7 25389015 PMC4231193

[2] Chopra M, Daviaud E, Pattinson R, et al. Saving the lives of South Africa’s mothers, babies, and children: Can the health system deliver? Lancet. 2009;374(9692):835–846. 10.1016/S0140-6736(09)61123-5 19709729

[3] Abdollahi F, Rezai Abhari F, Zarghami M. Post-partum depression effect on child health and development. Acta Med Iran. 2017;55(2):109–114. 28282707

[4] Ali NS, Mahmud S, Khan A, et al. Impact of postpartum anxiety and depression on child’s mental development from two peri-urban communities of Karachi, Pakistan: A quasi-experimental study. BMC Psychiatry. 2013;13:274. 10.1186/1471-244x-13-274 24148567 PMC3819469

[5] Goodman SH, Rouse MH, Connell AM, et al. Maternal depression and child psychopathology: A meta-analytic review. Clin Child Fam Psychol Rev. 2011;14(1):1–27. 10.1007/s10567-010-0080-1 21052833

[6] Makrides M, Gibson RA, McPhee AJ, et al. Effect of DHA supplementation during pregnancy on maternal depression and neurodevelopment of young children: A randomized controlled trial. JAMA. 2010;304(15):1675–1683. 10.1001/jama.2010.1507 20959577

[7] Parsons CE, Young KS, Rochat TJ, et al. Postnatal depression and its effects on child development: A review of evidence from low-and middle-income countries. Br Med Bull. 2012;101:57–79. 10.1093/bmb/ldr047 22130907

[8] Quevedo LA, Silva RA, Godoy R, et al. The impact of maternal post-partum depression on the language development of children at 12 months. Child Care Health Dev. 2012;38(3):420–424. 21651606 10.1111/j.1365-2214.2011.01251.x

[9] Netsi E, Pearson RM, Murray L, et al. Association of persistent and severe postnatal depression with child outcomes. JAMA Psychiatry. 2018;75(3):247–253. 10.1001/jamapsychiatry.2017.4363 29387878 PMC5885957

[10] Chen YH, Tsai SY, Lin HC. Increased mortality risk among offspring of mothers with postnatal depression: A nationwide population-based study in Taiwan. Psychol Med. 2011;41(11):2287–2296. 10.1017/s0033291711000584 21524332

[11] Wachs TD, Black MM, Engle PL. Maternal depression: A global threat to children’s health, development, and behavior and to human rights. Child Dev Perspect. 2009;3(1):51–59. 10.1111/j.1750-8606.2008.00077.x

[12] Coast E, Leone T, Hirose A, et al. Poverty and postnatal depression: A systematic mapping of the evidence from low and lower middle income countries. Health Place. 2012;18(5):1188–1197. 10.1016/j.healthplace.2012.05.004 22749341

[13] Mokwena K, Shiba D. Prevalence of postnatal depression symptoms in a primary health care clinic in Pretoria, South Africa: Management of health care services. Afr J Phys Health Educ Recreat Dance. 2014;20(Suppl. 1):116–127.

[14] Alkar ÖY, Gençöz T. Critical factors associated with early postpartum depression among Turkish women. Contemp Fam Ther. 2005;27:263–275. 10.1007/s10591-005-4043-5

[15] Gjerdingen D, McGovern P, Attanasio L, et al. Maternal depressive symptoms, employment, and social support. J Am Board Fam Med. 2014;27(1):87–96. 10.3122/jabfm.2014.01.130126 24390890 PMC3882899

[16] Guintivano J, Sullivan PF, Stuebe AM, et al. Adverse life events, psychiatric history, and biological predictors of postpartum depression in an ethnically diverse sample of postpartum women. Psychol Med. 2018;48(7):1190–1200. 10.1017/s0033291717002641 28950923 PMC6792292

[17] Iwata H, Mori E, Sakajo A, et al. Prevalence of postpartum depressive symptoms during the first 6 months postpartum: Association with maternal age and parity. J Affect Disord. 2016;203:227–232. 10.1016/j.jad.2016.06.002 27295378

[18] Fisher J, Mello MCD, Patel V, et al. Prevalence and determinants of common perinatal mental disorders in women in low-and lower-middle-income countries: A systematic review. Bull World Health Organ. 2012;90(2):139–149. 10.2471/BLT.11.091850 PMC3302553 22423165

[19] Prabhu S, George LS, Shyamala G, Noronha JA, Hebbar S. Prevalence and associated risk factors of postnatal depression in south asian region – A systematic review. Indian J Public Health Res Dev. 2019;10(5):329–333. 10.5958/0976-5506.2019.01022.2

[20] Redinger S, Norris SA, Pearson RM, et al. First trimester antenatal depression and anxiety: Prevalence and associated factors in an urban population in Soweto, South Africa. J Dev Orig Health Dis. 2018;9(1):30–40. 10.1017/s204017441700071x 28877770

[21] Canadian Paediatric Society. Maternal depression and child development. Paediatr Child Health. 2004;9(8):575–598. 10.1093/pch/9.8.575 19680490 PMC2724169

[22] Mokwena K, Masike I. The need for universal screening for postnatal depression in South Africa: Confirmation from a sub-district in Pretoria, South Africa. Int J Environ Res Public Health. 2020;17(19):6980. 10.3390/ijerph17196980 PMC7579387 32987674

[23] Novotney J, Maurer D. Is the Edinburgh postnatal depression scale an effective way to screen for postpartum depression? Evid Based Practice. 2017;20:E7–E8. 10.1097/01.EBP.0000541776.26600.35

[24] Maternal, Newborn, Child and Women’s Health (MNCWH). Strategic plan for maternal, newborn, child and women’s health (MNCWH) and nutrition in South Africa [homepage on the Internet]. 2012–2016 [cited 2021 Feb 01]. Available from: https://extranet.who.int/nutrition/gina/sites/default/filesstore/ZAF%202012%20MNCWHstratplan.pdf

[25] January J, Mutamba N, Maradzika J. Correlates of postnatal depression among women in Zimbabwean semi-urban and rural settings. J Psychol Afr. 2017;27(1):93–96. 10.1080/14330237.2016.1268299

[26] Villegas L, McKay K, Dennis CL, et al. Postpartum depression among rural women from developed and developing countries: A systematic review. J Rural Health. 2011;27(3):278–288. 10.1111/j.1748-0361.2010.00339.x 21729155

[27] Ashaba S, Kaida A, Coleman JN, et al. Psychosocial challenges facing women living with HIV during the perinatal period in rural Uganda. PLoS One. 2017;12(5):e0176256. 10.1371/journal.pone.0176256 28459866 PMC5411062

[28] Sawyer A, Ayers S, Smith H. Pre-and postnatal psychological wellbeing in Africa: A systematic review. J Affect Disord. 2010;123(1–3):17–29. 10.1016/j.jad.2009.06.027 19635636

[29] Tomlinson M, Cooper P, Stein A, et al. Post-partum depression and infant growth in a South African peri-urban settlement. Child Care Health Dev. 2006;32(1):81–86. 10.1111/j.1365-2214.2006.00598.x 16398794

[30] Stellenberg EL, Abrahams JM. Prevalence of and factors influencing postnatal depression in a rural community in South Africa. Afr J Prim Health Care Fam Med. 2015;7(1):1–8. 10.4102/phcfm.v7i1.874 PMC4729123 26842515

[31] Govender D, Naidoo S, Taylor M. Antenatal and postpartum depression: Prevalence and associated risk factors among adolescents’ in KwaZulu-Natal, South Africa. Depress Res Treat. 2020;2020:5364521. 10.1155/2020/5364521 32411457 PMC7204344

[32] Mbatha NL, Mokwena KE, Madiba S. Clinical and obstetric risk factors for postnatal depression in HIV positive women: A cross sectional study in health facilities in rural KwaZulu-Natal. Int J Environ Res Public Health. 2020;17(22):8425. 10.3390/ijerph17228425 PMC7697934 33202528

[33] Mokwena KE, Mbatha NL. Social and demographic factors associated with postnatal depression symptoms among HIV-positive women in primary healthcare facilities, South Africa. Healthcare (Basel). 2021;9(1):65. 10.3390/healthcare9010065 33445414 PMC7826739

[34] Peltzer K, Shikwane M. Prevalence of postnatal depression and associated factors among HIV-positive women in primary care in Nkangala district, South Africa. S Afr J HIV Med. 2011;12(4):24–28. 10.4102/sajhivmed.v12i4.168

[35] Rodriguez VJ, Matseke G, Cook R, et al. Infant development and pre- and post-partum depression in rural South African HIV-infected women. AIDS Behav. 2018;22(6):1766–1774. 10.1007/s10461-017-1925-0 28986652 PMC5889365

[36] Municipalities. Gauteng municipalities [homepage on the Internet]. 2021 [cited 2021 Aug 30]. Available from: https://municipalities.co.za/provinces/view/3/gauteng

[37] Municipalities. Free state municipalities [homepage on the Internet]. 2021 [cited 2021 Aug 30]. Available from: https://municipalities.co.za/provinces/view/2/free-state

[38] SouthAfricami. South Africa’s population density map [homepage on the Internet]. c2018 [updated 2020 July 27; cited 2022 Apr 24]. Available from: http://www.southafricanmi.com/population-density-map.html

[39] NDoH. Health [homepage on the Internet]. [cited 2022 Apr 24]. Available from: https://www.gov.za/about-sa/health#:~:text=Gauteng%20still%20comprises%20the%20largest,%25)%20living%20in%20this%20province

[40] Malakoane B, Heunis JC, Chikobvu P, et al. Public health system challenges in the Free State, South Africa: A situation appraisal to inform health system strengthening. BMC Health Serv Res. 2020;20(1):58. 10.1186/s12913-019-4862-y 31973740 PMC6979387

[41] Statistic South Africa. Provincial projection by sex and age (2002–2018). Statistics South Africa: 2018. [cited n.d.]. Available from: https://southafrica.opendataforafrica.org/topryig/provincial-projection-by-sex-and-age-2002-to-2018?province=1000000-south-africa

[42] RaosoftCom. Sample size calculator [homepage on the Internet]. 2004 [cited 2019 Dec]. Available from: http://www.raosoft.com/samplesize.html

[43] Abdollahi F, Lye M-S, Md Zain A, et al. Postnatal depression and its associated factors in women from different cultures. Iran J Psychiatry Behav Sci. 2011;5(2):5–11. PMID: ; PMCID: . 24644441 PMC3939973

[44] Smith-Nielsen J, Matthey S, Lange T, et al. Validation of the Edinburgh postnatal depression scale against both DSM-5 and ICD-10 diagnostic criteria for depression. BMC Psychiatry. 2018;18(1):393. 10.1186/s12888-018-1965-7 30572867 PMC6302501

[45] Shrestha SD, Pradhan R, Tran TD, et al. Reliability and validity of the Edinburgh postnatal depression scale (EPDS) for detecting perinatal common mental disorders (PCMDs) among women in low-and lower-middle-income countries: A systematic review. BMC Pregnancy Childbirth. 2016;16:72. 10.1186/s12884-016-0859-2 27044437 PMC4820998

[46] Javalkar SR, Vanitha SS. A study on postnatal depression among women attending tertiary care hospital in Davanagere, Karnataka. Natl J Community Med. 2018;9(3):167–171.

[47] Howard LM, Ryan EG, Trevillion K, et al. Accuracy of the whooley questions and the Edinburgh postnatal depression scale in identifying depression and other mental disorders in early pregnancy. Br J Psychiatry. 2018;212(1):50–56. 10.1192/bjp.2017.9 29433610 PMC6457164

[48] Judd F, Lorimer S, Thomson RH, et al. Screening for depression with the Edinburgh postnatal depression scale and finding borderline personality disorder. Aust N Z J Psychiatry. 2019;53(5):424–432. 10.1177/0004867418804067 30309241

[49] Brittain K, Mellins CA, Phillips T, et al. Social support, stigma and antenatal depression among HIV-infected pregnant women in South Africa. AIDS Behav. 2017;21(1):274–282. 10.1007/s10461-016-1389-7 PMC6116836 27052843

[50] Modjadji P, Mokwena K. Postnatal depression screening among postpartum women attending postnatal care at selected community health centres situated in the Nkangala District of South Africa. Open Public Health J. 2020;13(1):696–704. 10.2174/1874944502013010696

[51] Adamu AF, Adinew YM. Domestic violence as a risk factor for postpartum depression among Ethiopian women: Facility based study. Clin Pract Epidemiol Mental Health. 2018;14:109. 10.2174/1745017901814010109 PMC5971200 29997678

[52] Shamu S, Zarowsky C, Roelens K, et al. High-frequency intimate partner violence during pregnancy, postnatal depression and suicidal tendencies in Harare, Zimbabwe. Gen Hosp Psychiatry. 2016;38:109–114. 10.1016/j.genhosppsych.2015.10.005 26607330

[53] Ramchandani PG, Richter LM, Stein A, et al. Predictors of postnatal depression in an urban South African cohort. J Affect Disord. 2009;113(3):279–284. 10.1016/j.jad.2008.05.007 18571734

[54] Tsai AC, Tomlinson M, Comulada WS, et al. Intimate partner violence and depression symptom severity among South African women during pregnancy and postpartum: Population-based prospective cohort study. PLoS Med. 2016;13(1):e1001943. 10.1371/journal.pmed.1001943 26784110 PMC4718639

[55] January J, Chimbari MJ. Prevalence and factors associated with postnatal depression among women in two rural districts of Manicaland, Zimbabwe. S Afr J Psychiatr. 2018;24:1176–1176. 10.4102/sajpsychiatry.v24i0.1176 30473880 PMC6244063

[56] Bauer A, Knapp M, Parsonage M. Lifetime costs of perinatal anxiety and depression. J Affect Disord. 2016;192:83–90. 10.1016/j.jad.2015.12.005 26707352

[57] Kathree T, Petersen I. South African Indian women screened for postpartum depression: A multiple case study of postpartum experiences. S Afr J Psychol. 2012;42(1):37–50. 10.1177/008124631204200105

[58] Ogbo FA, Eastwood J, Hendry A, et al. Determinants of antenatal depression and postnatal depression in Australia. BMC Psychiatry. 2018;18:49. 10.1186/s12888-018-1598-x 29463221 PMC5819705

[59] Adeniyi OV, Ajayi AI, Moyaki MG, et al. High rate of unplanned pregnancy in the context of integrated family planning and HIV care services in South Africa. BMC Health Serv Res. 2018;18(1):140. 10.1186/s12913-018-2942-z 29482587 PMC5828463

[60] Aventin Á, Robinson M, Hanratty J, et al. PROTOCOL: Involving men and boys in family planning: A systematic review of the effective components and characteristics of complex interventions in low- and middle-income countries. Campbell Syst Rev. 2021;17(1):e1140. 10.1002/cl2.1140 PMC8356317 37050964

[61] Manikkam L, Burns JK. Antenatal depression and its risk factors: An urban prevalence study in KwaZulu-Natal. S Afr Med J. 2012;102(12):940–944. 10.7196/SAMJ.6009 23498042

[62] Dadi AF, Akalu TY, Baraki AG, et al. Epidemiology of postnatal depression and its associated factors in Africa: A systematic review and meta-analysis. PLoS One. 2020;15(4):e0231940. 10.1371/journal.pone.0231940 32343736 PMC7188237

[63] Mohamad Yusuff AS, Tang L, Binns CW, et al. Prevalence and risk factors for postnatal depression in Sabah, Malaysia: A cohort study. Women Birth. 2015;28(1):25–29. 10.1016/j.wombi.2014.11.002 25466643

[64] Shrivastava SR, Shrivastava PS, Ramasamy J. Antenatal and postnatal depression: A public health perspective. J Neurosci Rural Pract. 2015;6(1):116–119. 10.4103/0976-3147.143218 25552868 PMC4244771

[65] Al Dallal F, Grant I. Postnatal depression among Bahraini women: Prevalence of symptoms and psychosocial risk factors. EMHJ East Mediterranean Health J. 2012;18(5):439–445. 10.26719/2012.18.5.432 22764429

[66] Wittkowski A, Gardner PL, Bunton P, et al. Culturally determined risk factors for postnatal depression in Sub-Saharan Africa: A mixed method systematic review. J Affect Disord. 2014;163:115–124. 10.1016/j.jad.2013.12.028 24461216

[67] Docrat S, Besada D, Cleary S, et al. Mental health system costs, resources and constraints in South Africa: A national survey. Health Policy Plan. 2019;34(9):706–719. 10.1093/heapol/czz085 31544948 PMC6880339

[68] Andersson LMC, Twum-Antwi A, Staland-Nyman C, et al. Prevalence and socioeconomic characteristics of alcohol disorders among men and women in the Eastern Cape Province, South Africa. Health Soc Care Community. 2018;26(1):e143–e153. 10.1111/hsc.12487 28868804

[69] Davis EC, Rotheram-Borus MJ, Weichle TW, et al. Patterns of alcohol abuse, depression, and intimate partner violence among township mothers in South Africa over 5 years. AIDS Behav. 2017;21(Suppl. 2):174–182. 10.1007/s10461-017-1927-y PMC5839633 29027039

[70] Gibbs A, Dunkle K, Jewkes R. Emotional and economic intimate partner violence as key drivers of depression and suicidal ideation: A cross-sectional study among young women in informal settlements in South Africa. PLoS One. 2018;13(4):e0194885. 10.1371/journal.pone.0194885 29659595 PMC5901771

[71] De Yébenes Prous MAJG, Salvanés FR, Ortells LC. Validation of questionnaires. Reumatol Clín. 2009;5:171–177. (In English). 10.1016/S2173-5743(09)70115-7 21794604

[72] Lawrie TA, Hofmeyr GF, deJager M, Berk M. Validation of the Edinburgh postnatal depression scale on a cohort of South African women. SAMJ. 1998;88(10):1340–1344. 9807193

[73] Noonan M, Jomeen J, Doody O. A review of the involvement of partners and family members in psychosocial interventions for supporting women at risk of or experiencing perinatal depression and anxiety. Int J Environ Res Public Health. 2021;18(10):5396. 10.3390/ijerph18105396 34070148 PMC8158393

